# The association between *HDAC9* gene polymorphisms and stroke risk in the Chinese population: A meta-analysis

**DOI:** 10.1038/srep41538

**Published:** 2017-02-01

**Authors:** Xin Zhou, Tangming Guan, Shuyuan Li, Zinan Jiao, Xiaoshuang Lu, Xiaodi Huang, Yuhua Ji, Qiuhong Ji

**Affiliations:** 1 Institute of Immunology, College of Life Science and Technology, Jinan University, Guangdong 510630, China; 2 Affiliated Hospital of Guangdong Medical University, Zhanjiang 524001, China; 3 Department of Neurology, Affiliated Hospital of Nantong University, Nantong 226002, China

## Abstract

Several recent genome-wide association studies (GWASs) have suggested that the histone deacetylase 9 (*HDAC9*) gene is associated with stroke, but the reliability of these findings remains controversial, particularly for the data derived from different ethnicities and geographical locations. Therefore, we performed a meta-analysis to explore the associations between *HDAC9* polymorphisms and the risk of stroke in the Chinese population. All eligible case-control studies that met the search criteria were retrieved from multiple databases, and six case-control studies with a total of 2,356 stroke patients and 3,420 healthy controls were included. The pooled odds ratios (ORs) with 95% confidence intervals (95% CIs) were calculated to assess the strengths of the associations of 3 *HDAC9* gene polymorphisms with stroke risk. Our results revealed statistically significant associations of the rs2107595 (T/C) polymorphism with an increased risk of stroke in the allele, codominant and dominant models. Additionally, the rs2389995 (G/A) polymorphism was found to be significantly associated with a decreased risk of stroke in all genetic models. In conclusion, this meta-analysis suggested that the T allele of rs2107595 in *HDAC9* increases the risk of stroke but that the G allele of rs2389995 decreases the risk of stroke in the Chinese population.

Stroke is a leading cause of death and long-term adult disability throughout the world^1^. According to the estimates of the World Health Organization, nearly 15 million people suffer a stroke each year worldwide, and among these, there are approximately 5.5 million deaths and 5 million disabled people^2^. In China, the situation is even more serious, and the annual prevalence rate of stroke is approximately 170.3/100,000^3^. Stroke is a multi-factorial disorder that is associated with genetic and environmental factors^4^. Recently, several genome-wide association studies (GWASs) identified histone deacetylase 9 (*HDAC9*) as being significantly associated with ischaemic stroke^5^,^6^,^7^,^8^.

*HDAC9* is a member of the HDAC gene family that plays essential roles in the organization of chromatin structure and subsequent transcriptional regulation^9^. Different *HDAC9* polymorphisms have been reported to be associated with coronary artery disease (CAD)^7^,^10^, peripheral arterial disease (PAD)^11^, schizophrenia^12^, cancer^13^,^14^ and androgenetic alopecia^15^. Recently, the associations between *HDAC9* gene polymorphisms and the risk of stroke have been intensively investigated^5^,^6^,^7^,^8^. However, the community is still unable to reach a consensus, particularly regarding the data from different ethnic groups and geographical locations^5^,^16^. For example, the International Stroke Genetics Consortium (ISGC) and the Wellcome Trust Case Control Consortium 2 (WTCCC2) were the first to report that rs11984041 of *HDAC9* is significantly associated with large-vessel ischaemic stroke in European populations^5^. However, a recent study^16^ based on a Chinese Han population indicated that the rs11984041 of *HDAC9* is not polymorphic and claimed significant associations of rs2389995 and rs2240419, which are the other two SNPs of the *HDAC9* gene, with large-vessel stroke. Hence, we designed this meta-analysis to quantify the overall genetic effects of these three *HDAC9* polymorphisms on the risk of stroke in the Chinese population.

## Results

### Search results and study characteristics

**Figure 1:**
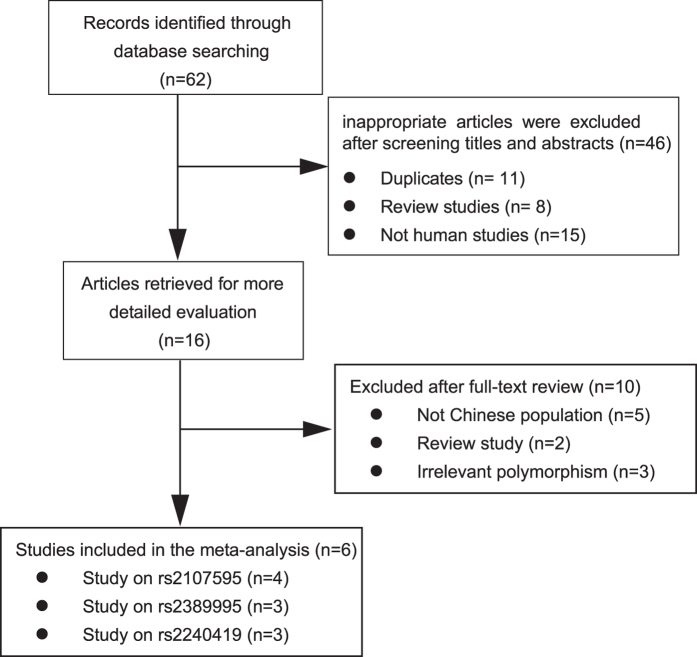
Flow diagram of the selection of eligible studies.

Figure 1 and Table 1 display the study selection process and main characteristics of the included studies, respectively. A total of 62 publications were identified in an initial search. After reviewing the titles and abstracts, 46 articles were excluded. The full texts of the remaining 16 studies were reviewed, 10 studies were excluded, and 2 studies^8^,^17^ on the SNP rs210759 of *HDAC9* were possibility of partial overlapping but this possible partial overlapping would not affect the final results (see Supplementary Tables S1 and S2), thus both of the two studies were included. Finally, 6 studies involving 2,356 cases and 3,420 controls were ultimately included in the present meta-analysis^8^,^16^,^17^,^18^,^19^,^20^. Regarding the rs2107595 (T/C) polymorphism, 4 studies^8^,^17^,^19^,^20^ were available and included a total of 1,812 cases and 2,136 controls. Regarding the rs2389995 (G/A) polymorphism, 3 studies^16^,^18^,^20^ involving a total of 1,045 cases and 1,798 controls were available. Regarding the rs2240419 (T/C) polymorphism, 3 studies^16^,^18^,^20^ involving a total of 1,054 cases and 1,798 controls were available. Additionally, the studied populations of these 6 papers were from 5 different locations in China, including Beijing, Shanghai, Heilongjiang, Gansu and Guangxi, and all of the participants were Han Chinese. Additionally, all of the included studies were of high quality, as indicated by the NOS scores of each study being above 5 points, and the genotype distributions in all of the controls were consistent with HWE.

### Power analysis

Before implementation of this meta-analysis, statistical power was assessed with the assumptions:α err prob = 0.05, OR = 1.20 (corresponding to a “weak to moderate” gene effect) for the three SNPs, and minor allele frequencies (MAF) of the 3 SNPs in the Chinese Han population in Beijing (HCB) were estimated from the 1000 Genomes. The present samples indicated that 96% power for rs2107595 (MAF = 0.301), 75% power for rs2389995 (MAF = 0.180), and 80% power for rs2240419 (MAF = 0.218). The power analysis indicated that these recruited samples could provide sufficient power in identifying the association between the 3 SNPs and stroke in the Chinese Han population.

### Quantitative synthesis

**Figure 2:**
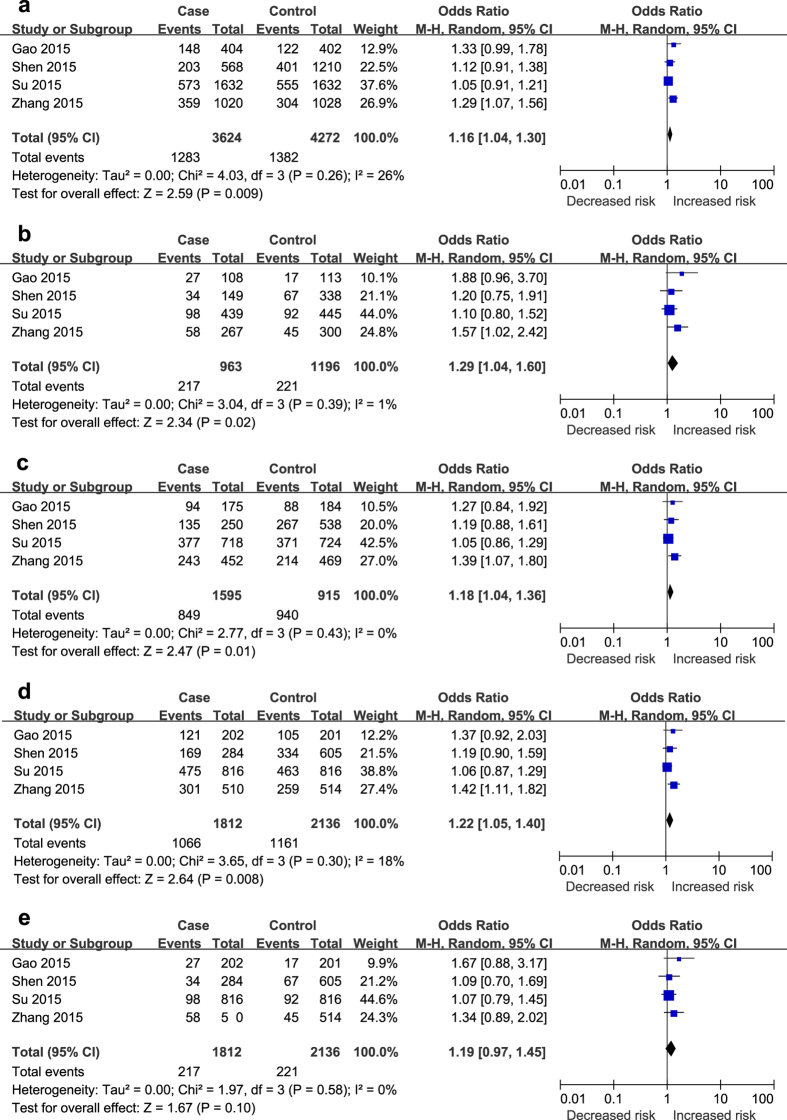
The associations of rs210759 with stroke in different genetic models. (**a**) Allele model (T vs. C). (**b**) Codominant model (TT vs. CC). (**c**) Codominant model (CT vs. CC). (**d**) Dominant model (TT + CT vs. CC). (**e**) Recessive model (TT vs. CC + CT).

**Figure 3:**
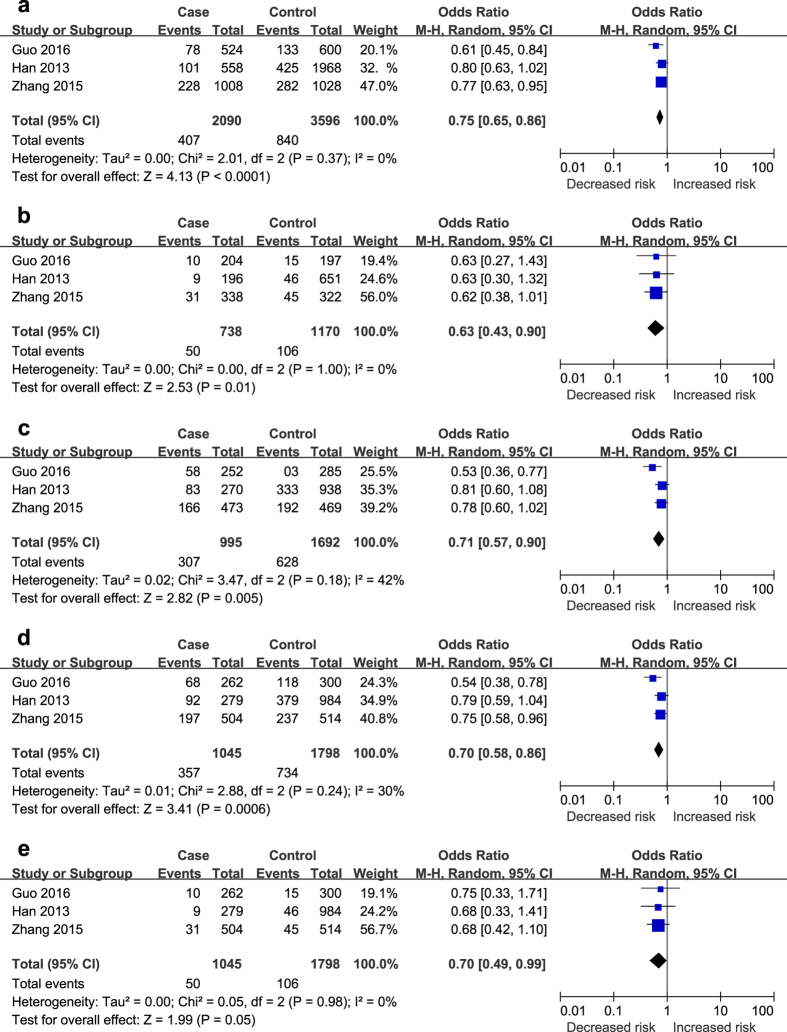
The associations of rs2389995 with stroke in different genetic models. (**a**) Allele model (G vs. A). (**b**) Codominant model (GG vs. AA). (**c**) Codominant model (AG vs. AA). (**d**) Dominant model (GG + AG vs. AA). (**e**) Recessive model (GG vs. AA + AG).

**Figure 4:**
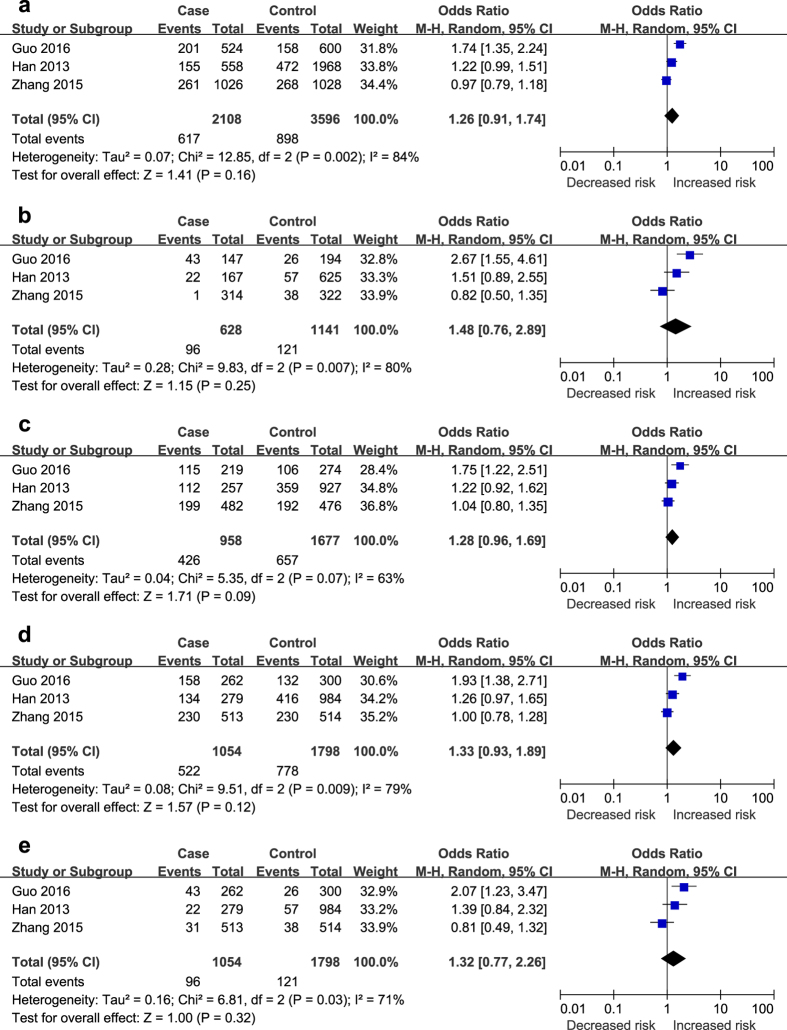
The associations of rs2240419 and stroke in the different genetic models. (**a**) Allele model (T vs. C). (**b**) Codominant model (TT vs. CC). (**c**) Codominant model (CT vs. CC). (**d**) Dominant model (TT + CT vs. CC). (**e**) Recessive model (TT vs. CC + CT).

As illustrated in Figs 2–4, different genetic models of rs2107595, rs2389995 and rs2240419 were used in our analysis.

The T allele of rs210759 was found to be significantly associated with an increased risk of ischaemic stroke. The following data were obtained: in the allele model, T vs. C, OR = 1.16, 95% CI: 1.04–1.30, and *P* < 0.01; in the codominant model, TT vs. CC, OR = 1.29, 95% CI: 1.04–1.60, *P* < 0.05; CT vs. CC, OR = 1.18, 95% CI: 1.04–1.36, and *P* < 0.05; in the dominant model, TT + CT vs. CC, OR = 1.22, 95% CI: 1.05–1.40 and *P* < 0.01; and the recessive model was not significant (TT vs. CC + CT, OR = 1.19, 95% CI: 0.97–1.45, *P* = 0.10) (Fig. 2).

The G allele of rs2389995 was found to be significantly associated with a decreased risk of ischaemic stroke in all models: allele (G vs. A, OR = 0.75, 95% CI: 0.65–0.86, *P* < 0.01); codominant (GG vs. AA, OR = 0.63, 95% CI: 0.43–0.90, *P* < 0.05; AG vs. AA, OR = 0.71, 95% CI: 0.57–0.90, *P* < 0.01); dominant (GG + AG vs. AA, OR = 0.70, 95% CI: 0.58–0.86, *P* < 0.01); and recessive (GG vs. AA + AG, OR = 0.70, 95% CI: 0.49–0.99, *P* = 0.05) (Fig. 3).

However, no significant relationship of the rs2240419 polymorphism with IS risk was found in any of the four genetic models (T vs. C: OR = 1.26, 95% CI = 0.91–1.74, *P* = 0.16; TT vs. CC: OR = 1.48, 95% CI = 0.76–2.89, *P* = 0.25; CT vs. CC: OR = 1.28, 95% CI = 0.96–1.69, *P* = 0.09; TT + CT vs. CC: OR = 1.33, 95% CI = 0.93–1.89, *P* = 0.12; and TT vs. CT + CC: OR = 1.32, 95% CI = 0.77–2.26, *P* = 0.32). Remarkable between-study heterogeneity was present, and thus a random-effects model was used to calculate the pooled estimates Fig. 4).

### Sensitivity analysis

**Figure 5:**
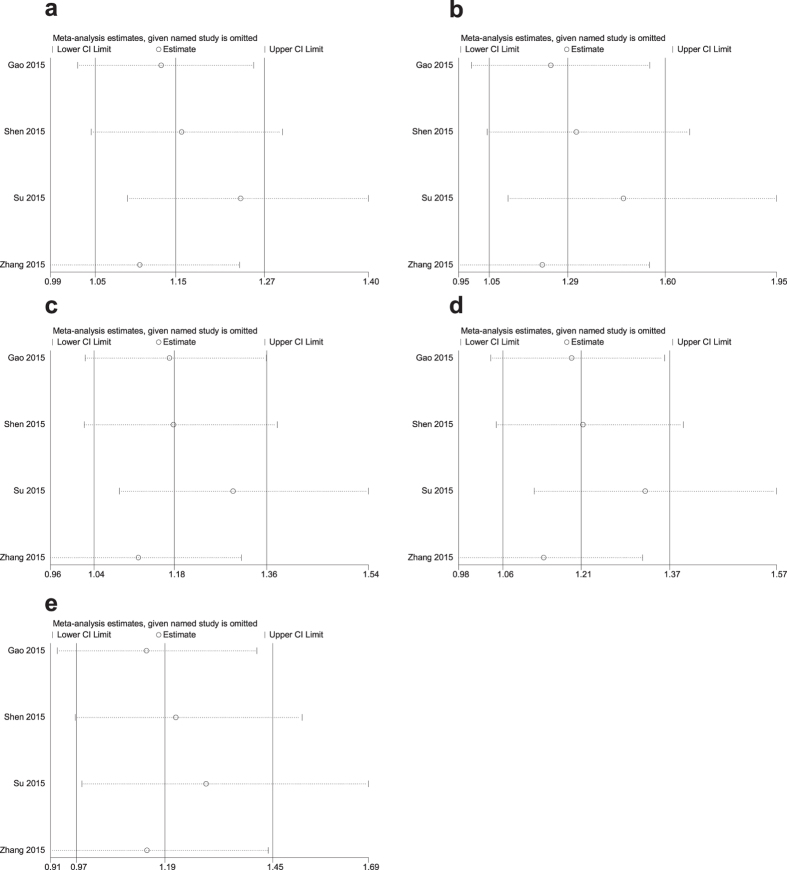
Sensitivity analysis of the association of rs210759 and stroke in different genetic models. (**a**) Allele model (T vs. C). (**b**) Codominant model (TT vs. CC). (**c**) Codominant model (CT vs. CC). (**d**) Dominant model (TT + CT vs. CC). (**e**) Recessive model (TT vs. CC + CT).

**Figure 6:**
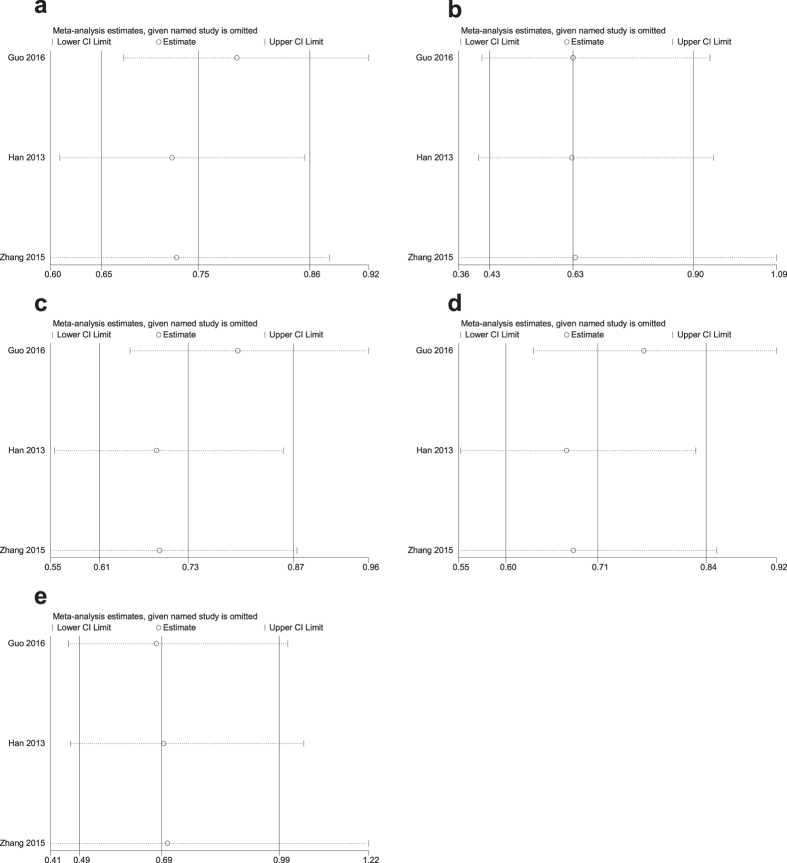
Sensitivity analysis of the association of rs2389995 and stroke in the different genetic models. (**a**) Allele model (G vs. A). (**b**) Codominant model (GG vs. AA). (**c**) Codominant model (AG vs. AA). (**d**) Dominant model (GG + AG vs. AA). (**e**) Recessive model (GG vs. AA + AG).

**Figure 7:**
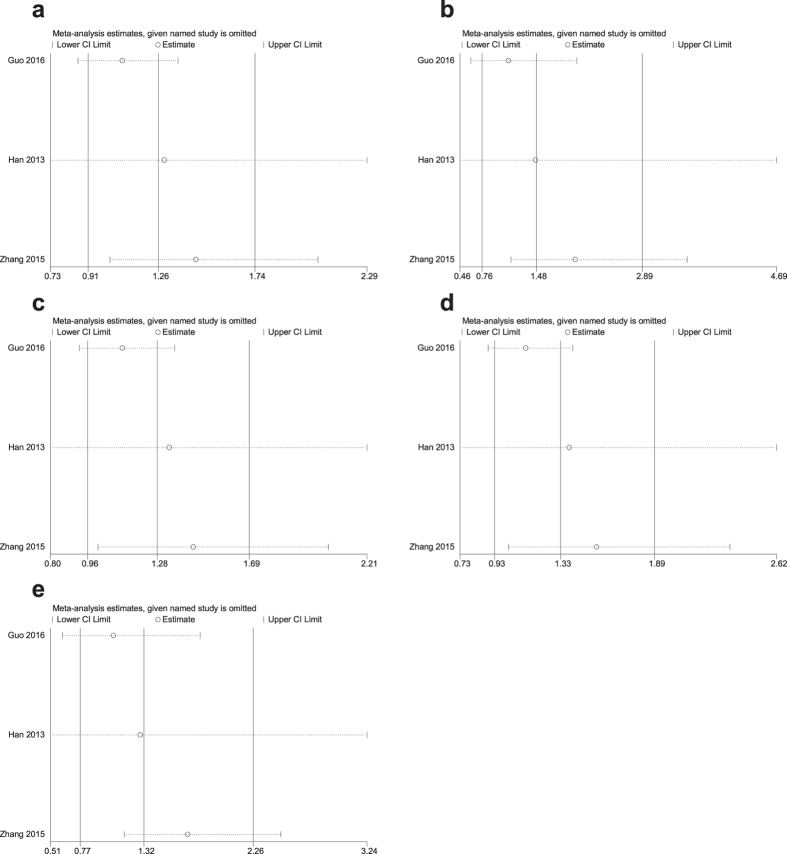
Sensitivity analysis of the association of rs2240419 and stroke in the different genetic models. (**a**) Allele model (T vs. C). (**b**) Codominant model (TT vs. CC). (**c**) Codominant model (CT vs. CC). (**d**) Dominant model (TT + CT vs. CC). (**e**) Recessive model (TT vs. CC + CT).

A sensitivity analysis of the summary odds ratios of the relationships of the three SNPs and the risk of stroke was conducted by sequentially, omitting each study. The corresponding pooled ORs were not significantly altered after excluding each eligible study (Figs 5–7).

### Publication bias

**Figure 8:**
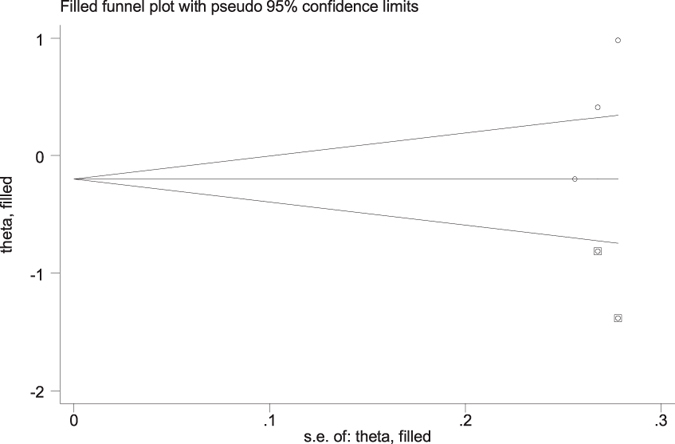
Funnel plot after filling 2 studies using the trim and fill method in the codominant model (TT vs. CC) of rs2240419.

No evidence of publication bias was detected regarding the ORs of the three SNPs in this study by either Begg’s or Egger’s test, with the exception of the codominant model of rs2240419 (TT vs. CC: Begg’s test *P* = 0.30, Egger’s test *P* = 0.01). There were no significant differences for the pooled ORs before or after using the “trim and fill” method in the codominant model (TT vs. CC) of rs2240419 (Table 2 and Fig. 8).

## Discussion

Recently, several GWASs identified a novel association between *HDAC9* and ischaemic stroke. In China, Han Y^16^ and Guo QX^18^ found that the SNPs rs2389995 and rs2240419 are significantly associated with stroke risk in the Chinese Han population. Similarly, Zhang *et al*.^20^ demonstrated that three SNPs (rs2107595, rs2389995 and rs2240419) of *HDAC9* are significantly associated with an increased risk of stroke in a northwest Chinese Han population. However, another two studies^8^,^17^ revealed that the *HDAC9* polymorphism loci rs2107595 may be not associated with stroke risk in southern Han Chinese. Due to relatively small samples from different populations, these studies demonstrated inconsistent results. Therefore, we performed a meta-analysis to estimate the association between the three SNPs (rs2107595, rs2389995 and rs2240419) of *HDAC9* and the stroke risk in the Chinese Han population. To the best of our knowledge, this is the first meta-analysis to explore the relationships between *HDAC9* gene polymorphisms and stroke susceptibility in a Chinese population. Six case-control studies^8^,^16^,^17^,^18^,^19^,^20^ with a total of 2,356 stroke patients and 3,420 healthy controls were included in our meta-analysis, which was sufficiently powered to detect stroke risk associated with *HDAC9 gene* polymorphisms.

In our meta-analysis, the main finding was that the T allele of rs2107595 confers the risk of stroke in all the three models (i.e., the allele, codominant and dominant models) in the Chinese population, whereas the G allele of rs2389995 decreases the risk of ischaemic stroke in all of the comparison models, and no significant association of the rs2240419 T/C polymorphism and the risk of stroke was observed. Our results have some differences from the previous studies. A possible explanation for this phenomenon is that the previous single studies of stroke had small samples size, and thus the significance of current work may not be justified; thus, further studies are needed to clarify the effects of the 3 SNPs on the risk of stroke. Furthermore, the differential allele frequencies of the 3 SNPs exerted disproportionate levels of influence on the stroke risks in different populations. For example, the minor allelic frequencies (MAFs) of the 3 SNPs rs2107595, rs2389995 and rs2240419 of *HDAC9* are 0.301, 0.180 and 0.218, respectively, in the Chinese Han population in Beijing (HCB), whereas the MAFs are 0.367 0.210 and 0.290, respectively, in the southern Han Chinese population (CHS) based on the data from the 1000 G. Besides, the MAFs of the 3 SNPs rs2107595, rs2389995 and rs2240419 are 0.168, 0.006 and 0.244, respectively, in the European population (EUR), and it may be one possible reason for the inconsistent result between the Chinese and the European. Additionally, the linkage disequilibrium patterns in alleles between the different ethnic populations might also contribute to this phenomenon, for instance, the low linkage disequilibrium between the rs2389995 and rs2240419 has been found in the Chinese Han population in Beijing (HCB), as indicated by an r^2^ value of 0.059 and a D’value of 0.274, whereas an r^2^ value of 0.108 and a D’value of 0.409 was described in the southern Han Chinese population (CHS) based on the data from the 1000 G. Finally, significant heterogeneity was observed among all genetic models of rs2240419, and factors, such as genotyping method, subtype of stroke, gender distribution, personal history, past medical history and other, might be potential sources of heterogeneity.

Additionally, the genotype distributions of the controls did not deviate from HWE in any study. The NOS results indicated that the included studies were credible. Moreover, sensitivity analysis was conducted, and it did not significantly alter the combined ORs. Additionally, no evidence of publication bias was identified by either Begg’s or Egger’s tests with the exception of the codominant model of rs2240419. However, there was no significant difference for pooled ORs before and after using the ‘trim and fill’ method in the codominant model of rs2240419. Taken together, the results of this meta-analysis are reliable and stable.

There were some limitations in the current study. First, the sample sizes of the studies included in our meta-analysis were relatively small, which made it difficult to perform subgroup analysis to evaluate the heterogeneity among the included studies. Second, the heterogeneity detected in all genetic models of rs2240419 might have affected the precision of outcome even though we used the random-effects model to calculate pool ORs. Third, stroke is a multi-factorial disorder that is associated with genetic and environmental factors, such as diet, life style, and climate. However, none of the original studies accounted for gene-environment interactions. Further studies are needed to clarify whether these environmental factors affected the polymorphism of *HDAC9* and subsequent strokes. Fourth, only articles in English and Chinese language were included; thus, studies of other ethnicities and those written in other languages were neglected. Finally, three non-English (Chinese) articles were included in this analysis. Although inclusion of non-English articles may be difficult for non-native speakers to read and understand, it is essential for ensuring the generalizability of systematic analysis.

It is now accepted that the SNPs within the *HDAC9* gene on chromosome 7p21.1 are implicated in stroke risk^5^; however, the underlying mechanism by which variants in the *HDAC9* region are associated with the risk of stroke is still unclear. Recent studies have suggested that a deficiency of the *HDAC9* gene attenuates atherosclerosis^10^,^21^ and that *HDAC9* can increase risk by altering brain ischaemic responses and neuronal survival^5^. Therefore, the risk alleles in this region are potentially involved in stroke risk via the modulation of *HDAC9* expression. Further studies are required to clarify the roles of these variants in ischaemic stroke.

In summary, our meta-analysis suggested that the T allele of rs2107595 in the *HDAC9* gene increases the risk of stroke in the Chinese population, whereas the minor G allele of rs2389995 may be associated with a decreased risk of stroke. No association of stroke risk with the rs2240419 polymorphism was identified. Due to the above-mentioned limitations, a well-designed large-scale study that includes ethnicities and considers both genetic and environmental factors is required to confirm and expand these findings.

## Materials and Methods

### Search strategy

In accordance with the Preferred Reporting Items for Systematic Reviews and Meta-analyses (PRISMA) statement^22^, we searched the related literature of the electronic records of the PubMed, Science Direct, Chinese National Knowledge Infrastructure (CNKI), WANFANG, and VIP databases published through 1 April 2016. The search terms included the following key words: (“histone deacetylase 9” OR “*HDAC9*”) AND (“polymorphism” OR “gene” OR “allele” OR “genetics” OR “variant” OR “SNP” OR “mutation”) AND (“stroke” OR “cerebral ischaemic” OR “brain infarction” OR “ischaemic stroke cerebrovascular disease”). Furthermore, the references of selected articles and the abstracts presented at related conferences were also checked by hand to identify additional potential studies. The languages were limited to English and Chinese.

### Inclusion criteria

The inclusion criteria for the studies were as follows: (1) studies of the association between *HDAC9* gene polymorphisms and ischaemic stroke cerebrovascular disease in Chinese populations; (2) studies in which the diagnoses of IS were confirmed by computed tomographic (CT) or magnetic resonance imaging (MRI); (3) case-control studies; (4) the genotype distributions in both the cases and controls were available to calculate the OR and 95% CI; and (5) the genotype distribution in control group was consistent with Hardy-Weinberg equilibrium (HWE). Additionally, we excluded reviews, abstracts, and redundant and animal studies.

### Data extraction

Two independent investigators extracted the relevant data from all included studies based on the inclusion criteria, and a third investigator verified the data. The following information from eligible studies was extracted: the first author’s name, publication year, ethnicity, geographical location (province or city), sample size, genotyping method, and the *HDAC9* genotype distributions and alleles in the case and control groups.

### Quality assessment

Two investigators independently assessed the qualities of the included studies in accordance with the Newcastle-Ottawa scale (NOS)^23^, which is based on three aspects: selection, comparability, and exposure. Studies with scores of 5 points or higher were considered to be of high quality.

### Statistical analysis

The associations of the rs2107595, rs2389995, and rs2240419 polymorphisms with the risk of stroke were assessed by the pooled ORs with the corresponding 95% CIs under the following genetic models: allele model, codominant model, dominant model, and recessive model. The heterogeneity between studies was determined by the Cochrane’s Q-statistic test^24^, and the inconsistency was quantified with the I^2^ statistic. When *I*^*2*^ > 50% or *P*_*Q*_ ≤ 0.1, which suggest substantial heterogeneity, a random-effects model (DerSimonian-Laird method)^25^ was used; otherwise, the fixed-effects model (Mantel-Haenszel method)^26^ was applied. The Hardy-Weinberg equilibria (HWE) of the genotype distributions in the controls of the eligible studies were examined with Pearson’s χ^2^ test, *P* < 0.05 was considered statistically significant. The power analysis was calculated by using the Power and Sample Size Program software^27^. Sensitivity analysis was conducted by sequentially omitting each study to validate the reliability of the results. Publication bias was examined with Begg’s funnel plot and Egger’s test^28^, and *P* < 0.05 was considered statistically significant. If publication bias existed, the non-parametric ‘trim and fill’ method was used to adjust for the bias. All analyses were conducted using the RevMan 5.1 and STATA 12.0 software packages.

## Additional Information

**How to cite this article:** Zhou, X. *et al*. The association between *HDAC9* gene polymorphisms and stroke risk in the Chinese population: A meta-analysis. *Sci. Rep.* **7**, 41538; doi: 10.1038/srep41538 (2017).

**Publisher's note:** Springer Nature remains neutral with regard to jurisdictional claims in published maps and institutional affiliations.

## Supplementary Material

Supplementary Table S1

Supplementary Table S2

## Figures and Tables

**Table 1:** Characteristics of the studies included in the meta-analysis.

Author	Year	Study design	Control source	Genotype distribution (case/control)			HWE (*P*)	NOS
**SNP rs2107595**				**TT**	**TC**	**CC**		
Gao M	2015	Case-control	Hospital based	27/17	94/88	81/96	0.61	6
Shen TT	2015	Case-control	Hospital based	34/67	135/267	115/271	0.92	7
Su L	2015	Case-control	Hospital based	98/92	377/371	341/353	0.71	7
Zhang ZC	2015	Case-control	Hospital based	58/45	243/214	209/255	0.99	8
**SNP rs2389995**				**GG**	**GA**	**AA**		
Guo QX	2016	Case-control	Hospital based	10/15	58/103	192/182	0.93	7
Han Y	2013	Case-control	Population based	9/46	83/333	187/605	0.98	8
Zhang ZC	2015	Case-control	Hospital based	31/45	166/192	307/277	0.16	8
**SNP rs2240419**				**TT**	**TC**	**CC**		
Guo QX	2016	Case-control	Hospital based	43/26	115/106	104/168	0.12	7
Han Y	2013	Case-control	Population based	22/57	112/359	145/568	0.98	8
Zhang ZC	2015	Case-control	Hospital based	31/38	199/192	283/284	0.48	8

**Table 2:** Publication bias tests of the associations of rs2107595, rs2389995 and rs2240419 polymorphisms with stroke.

Comparisons	Egger test			Begg test
	Coefficient	*P* value	95% CI	*P* value
**SNP rs2107595** (**T**/**C**)				
T vs. C	2.96	0.29	(−6.07, 11.99)	0.73
TT vs. CC	2.82	0.19	(−3.47, 9.12)	0.31
TC vs. CC	1.83	0.48	(−7.31, 10.97)	0.73
TT + TC vs. CC	2.43	0.40	(−7.31, 12.18)	0.73
TT vs. TC + CC	2.31	0.17	(−2.48, 7.10)	0.31
**SNP rs2389995** (**G**/**A**)				
G vs. A	−3.99	0.39	(−39.34, 31.37)	1.00
GG vs. AA	0.07	0.49	(−0.81, 0.96)	1.00
GA vs. AA	−6.87	0.23	(40.49, 26.75)	1.00
GG + GA vs. AA	−5.92	0.30	(−44.94, 33.11)	1.00
GG vs. GA + AA	0.35	0.53	(−4.55, 5.26)	0.30
**SNP rs2240419** (**T**/**C**)				
T vs. C	20.38	0.10	(−21.71, 62.47)	0.30
TT vs. CC	54.16	0.01	(47.39, 60.93)	0.30
TC vs. CC	9.84	0.07	(−4.82, 24.49)	0.30
TT + TC vs. CC	13.89	0.10	(−12.58, 40.36)	0.30
TT vs. TC + CC	64.41	0.11	(−73.05, 201.86)	0.30
