# Supplementary material for: The association between HDAC9 gene polymorphisms and stroke risk in the Chinese population: A meta-analysis
Source: Sci Rep. 2017 Feb 1;7:41538. doi: 10.1038/srep41538 (PMC5286403; doi:10.1038/srep41538)
Supplement: Supplementary Table S2 [file srep41538-s2.pdf]

The association between *HDAC9* gene polymorphisms and stroke risk in the Chinese population: A meta-analysis

Xin Zhou[1], Tangming Guan[2], Shuyuan Li[1], Zinan Jiao[1], Xiaoshuang Lu[1], Xiaodi Huang[1], Yuhua Ji[1*], QiuhongJi[3*]

Supplementary Table S2. The result of the association between HDAC9 rs210759(T/C) polymorphisms and stroke after removing the possible overlapping samples(Shen TT's study).

Gene polymorphism	Number of studies	Genetic model	OR	95%CI	*P* value
SNP rs2107595(T/C)	3	T v.s C	1.16	1.04-1.29	< 0.01
		TT v.s CC	1.32	1.04-1.68	< 0.05
		CT v.s CC	1.18	1.02-1.37	< 0.05
		TT+CT v.s CC	1.21	1.05-1.40	< 0.01
		TT vs CC CT	1.22	0.97-1.53	0.09
